# 1,25 dihydroxyvitamin D-mediated orchestration of anticancer, transcript-level effects in the immortalized, non-transformed prostate epithelial cell line, RWPE1

**DOI:** 10.1186/1471-2164-11-26

**Published:** 2010-01-13

**Authors:** Pavlo L Kovalenko, Zhentao Zhang, Min Cui, Steve K Clinton, James C Fleet

**Affiliations:** 1 Department of Foods and Nutrition and the Interdepartmental Nutrition Program, Purdue University, West Lafayette, IN 47907-2059 USA; 2 Department of Internal Medicine, Division of Hematology and Oncology and the Comprehensive Cancer Center, The Ohio State University, Columbus, OH 43210 USA

## Abstract

**Background:**

Prostate cancer is the second leading cause of cancer mortality among US men. Epidemiological evidence suggests that high vitamin D status protects men from prostate cancer and the active form of vitamin D, 1α,25 dihydroxyvitamin D_3 _(1,25(OH)_2_D) has anti-cancer effects in cultured prostate cells. Still, the molecular mechanisms and the gene targets for vitamin D-mediated prostate cancer prevention are unknown.

**Results:**

We examined the effect of 1,25(OH)_2_D (+/- 100 nM, 6, 24, 48 h) on the transcript profile of proliferating RWPE1 cells, an immortalized, non-tumorigenic prostate epithelial cell line that is growth arrested by 1,25(OH)_2_D (Affymetrix U133 Plus 2.0, n = 4/treatment per time and dose). Our analysis revealed many transcript level changes at a 5% false detection rate: 6 h, 1571 (61% up), 24 h, 1816 (60% up), 48 h, 3566 (38% up). 288 transcripts were regulated similarly at all time points (182 up, 80 down) and many of the promoters for these transcripts contained putative vitamin D response elements. Functional analysis by pathway or Gene Set Analysis revealed early suppression of WNT, Notch, NF-kB, and IGF1 signaling. Transcripts related to inflammation were suppressed at 6 h (e.g. IL-1 pathway) and suppression of proinflammatory pathways continued at later time points (e.g. IL-17 and IL-6 pathways). There was also evidence for induction of anti-angiogenic pathways and induction of transcripts for protection from oxidative stress or maintenance of cell redox homeostasis at 6 h.

**Conclusions:**

Our data reveal of large number of potential new, direct vitamin D target genes relevant to prostate cancer prevention. In addition, our data suggests that rather than having a single strong regulatory effect, vitamin D orchestrates a pattern of changes within prostate epithelial cells that limit or slow carcinogenesis.

## Background

Several population-based studies have shown that low UV exposure or low plasma vitamin D metabolite levels increase prostate cancer risk [1-3]. The hormonal form of vitamin D, 1α,25-dihydroxyvitamin D_3 _(1,25(OH)_2_D) or its analogs have anti-cancer effects in cancer cells or animal tumor models that may be mediated through multiple mechanisms including inducing growth arrest, promoting cell differentiation, lowering apoptotic thresholds, and suppressing angiogenesis or metastasis (for current review see [4]). In prostate cancer cells, the growth inhibitory actions of 1,25(OH)_2_D require the presence of the vitamin D receptor (VDR), a ligand-inducible transcription factor [5-7]. However, it is not clear whether the chemopreventative effect of high vitamin D status in the normal, healthy prostate is mediated by the same mechanisms.

Many vitamin D target genes have been identified and characterized in the context of vitamin D's traditional actions in the control of calcium metabolism [8]. In contrast, very few 1,25(OH)_2_D-regulated gene targets have been definitively identified in the context of prostate cancer, much less normal prostate biology. For example, 1,25(OH)_2_D directly induces transcription of the cyclin dependent kinase inhibitor gene p21 in U937 leukemia cells [9]. However, in LNCaP human prostate carcinoma cells 1,25(OH)_2_D mediated accumulation of p21 mRNA appears to be indirect [10] through induction of IGF binding protein 3 (IGFBP-3) gene expression and suppression of IGF-1 signaling [11]. A number of candidate vitamin D target genes have been identified in other cell systems but it is not clear if they are relevant to prostate cancer prevention. For example, in breast cancer cells the 1,25(OH)_2_D analog EB1089 up-regulates expression of TGFβ_1 _and β_2 _mRNA [12] and down regulates the anti-apoptotic protein bcl-2 [13], while in breast, ovarian, and neuroblastoma cells, c-myc has been identified as a target of 1,25(OH)_2_D-mediated transcriptional repression [14,15]. In addition, gene expression profiling of EB1089 action in squamous carcinoma cells [16,17] shows that 1,25(OH)_2_D modulates expression of transcripts encoding extracellular matrix proteins, cell adhesion proteins, DNA repair enzymes, and factors controlling oxidative stress. These data suggest that the cancer preventive impact of 1,25(OH)_2_D may utilize unique mechanisms in different tissues or that 1,25(OH)_2_D impacts multiple pathways involved in carcinogenesis.

cDNA microarray analysis has been used on both human primary prostate epithelial cells and prostate cancer cells to identify potential target genes of 1,25(OH)_2_D [18-22]. However, these earlier studies have limitations that prevent their results from being applied more generally, e.g. they lack the sample replication that permits statistical analysis with sufficient power. In this study we examined 1,25(OH)_2_D induced changes in the transcriptome of the phenotypically normal, immortalized human prostate epithelial cell line RWPE1. These findings provide new insight into the mechanisms that may be used by vitamin D to prevent the development of human prostate cancer.

## Results

### Time course analysis of 1,25(OH)_2_D induced genes

Using a 5% FDR cut-off, we identified 5435 transcripts as significantly differentially expressed in at least one time point (Table 1). Following treatment with 1,25(OH)_2_D the number of differentially expressed transcripts was increased over time from 1571 at 6 h to 3566 at 48 h. At 6 and 24 h, the transcripts were predominantly up-regulated (60.7% and 59.6%) while at the 48 h time point the transcripts were predominantly down-regulated (62.3%). 1,25(OH)_2_D treatment significantly altered the expression of 288 transcripts at all three time points; 262 of these changed in the same direction and 63.2% were up-regulated. Although many of our transcript-level changes were greater than 1.5-fold, our use of sample replicates, quality controls, and careful statistical analysis allowed us to see more subtle changes that may have biological relevance. A detailed list of all significantly differentially expressed transcripts is available in Additional File 1. The entire list of 25,986 transcripts analyzed and their FDR value is available upon request.

**Table 1:** Transcripts that were significantly differentially expressed (5% FDR) in RWPE1 cells after treatment with 1,25(OH)_2_D.

	Up (>1.5×)	Down (>1.5×)	Total (>1.5×)
**6 h**	954(696)	617(340)	1571(1036)
**24 h**	1083(371)	733(243)	1816(614)
**48 h**	1343(410)	2223(639)	3566(1049)
Any time point *	2537(1076)	3009(956)	5435(2012)
All time points *	182(115)	80(37)	262(152)

*"Any time point" indicates transcripts that were regulated in at least one time point. "All time points" indicates transcripts that were differentially expressed in the same direction at all three time points.

### RT-PCR and ChIP confirmation of 1,25(OH)_2_D regulation

Eleven transcripts identified in the microarray analysis as 1,25(OH)_2_D induced were selected for PCR validation. These were selected based upon three criterion: they are classic vitamin D target genes (i.e. CYP24, TRPV6), they had been identified in other studies (e.g. TXNRD1, IGFBP3, P2RY2), and they spanned a wide range of expression levels (i.e. CYP24, CD14, TXNRD1, IGFBP3, P2RY2, CYP26B1, SEMA3B, SEMA3F were all up-regulated while VAV3, AKAP12, and APCDD1 were suppressed, Table 2).

**Table 2:** RT-PCR validation of expression values from microarray analysis for selected genes in primary human prostate epithelial cells (hPEC), and LNCaP cells.

	RWPE1 - Microarray (n = 4)			hPEC(n = 3)			LNCaP (n = 3)
Gene	6 h	24 h	48 h	#1	#2	#3	8 h
**CYP24**	1072*	73.9*	63.2*	231 ± 5*	790 ± 192*	959 ± 184*	39.2 ± 2.6*
**CD14**	226*	27.6*	27.4*	6.3 ± 0.8*	19.6 ± 1.3*	33.8 ± 8.0*	1.6 ± 0.3
**TXNRD1**	7.1*	1.1	1.5*	2.1 ± 0.2*	6.0 ± 0.1*	9.6 ± 1.8*	4.1 ± 0.9*
**IGFBP3**	3.0*	-2.7*	-6.4*	3.1 ± 0.4*	38.0 ± 3.1*	22.0 ± 4.8	2.9 ± 0.3*
**P2RY2**	13.5*	3.8	2.9*	1.6 ± 0.1*	5.2 ± 1.0	4.3 ± 0.2*	1.3 ± 0.1
**CYP26B1**	5.6*	1.0	1.0	-1.1 ± 0.1	-1.2 ± 0.3	-1.3 ± 0.1	2.7 ± 0.1*
**SEMA3B**	19.5*	13.4*	18.5*	4.1 ± 0.2*	2.5 ± 0.6	5.7 ± 0.3*	2.2 ± 0.4*
**SEMA3F**	2.5*	1.5*	1.7*	1.1 ± 0.2	-1.4 ± 0.2	-1.3 ± 0.1	1.8 ± 0.2*
**VAV3**	-2.7*	-2.3*	-1.4*	-1.3 ± 0.1	-1.2 ± 0.2	-1.3 ± 0.3	1.4 ± 0.1
**APCDD1**	-5.0*	-2.3*	-2.6*	-3.1 ± 0.2	-1.5 ± 0.3	-1.6 ± 0.3	3.2 ± 0.1*
**AKAP12**	9.9*	1.0	1.0	4.1 ± 0.8*	5.1 ± 1.8*	5.3 ± 1.9	7.1 ± 1.3
**TRPV6**	A^#^	A^#^	A^#^	23.3 ± 5.0*	86.0 ± 20.6*	153 ± 40.6*	4.6 ± 0.1*

Data are expressed as mean fold change +SEM of GAPDH-normalized expression (in arbitrary units).

* p < 0.05 (RT-PCR) or FDR<5% (Microarray data), # Absent

**Figure 1:**
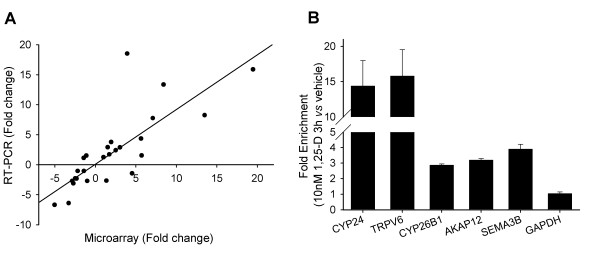
**Real Time-PCR and ChIP validation of differential regulation of selected transcripts**. **(A) Correlation between Microarray and Real Time-PCR data**. 9 transcripts identified as differentially expressed by microarray (TXNRD1, IGFBP3, P2RY2, Cyp26B1, SEMA3B, SEMA3F, VAV3, AKAP12 and APCDD1) were examined by Real Time-PCR for 1,25(OH)_2_D-induced changes in expression at the 6, 24, and 48 h. Fold changes of RT-PCR validated transcripts were compared across their fold change identified in microarray analysis. The regression line was defined by the following equation: PCR fold change = 0.91(Microarray data fold change) + 0.09; r^2 ^= 0.64. **(B) ChIP assays of VDR recruitment to putative VDR binding sites. **RWPE1 cells were treated with vehicle or 10 nM 1,25(OH)_2_D for 3 h. DNA precipitates were measured with RT-PCR using primers spanning known VDREs (CYP24, TRPV6 and SEMA3B) and predicted VDREs (CYP26B1 and AKAP12). The results are shown as mean ± SEM (n = 3).

With the exception of CYP24 and CD14, which were higher in the array data, and TRPV6, which was "absent" in the array data, the expression of transcripts was similar between the array and RT-PCR analysis in vitamin D-treated RWPE1 cells (Figure 1A). We also examined the 1,25(OH)_2_D-mediated expression of these in other prostate cell lines to determine if their regulation was a generalizable response. A similar effect of 1,25(OH)_2_D on these transcripts was observed cultures of primary human prostate epithelial cells, but a blunted response to 1,25(OH)_2_D treatment was seen in LNCaP cells (Table 2).

Potential VDR binding sites were identified in the genes for nine 1,25(OH)_2_D-induced transcripts using a bioinformatic approach. The binding of VDR to the promoter areas of five of these genes was demonstrated by ChIP analysis in 1,25(OH)_2_D-treated RWPE1 cells (CYP24, TRPV6, CYP26B1, AKAP12 and SEMA3B, Figure 1B) and the extent of their enrichment was consistent with the 1,25(OH)_2_D-induced accumulation of their transcripts. This demonstrates that differential regulation of transcript levels can be both direct (i.e. demonstrated by VDR binding) and indirect (i.e. no binding in a ChIP assay).

### Cluster analysis for identification of groups with similar patterns of expression

**Figure 2:**
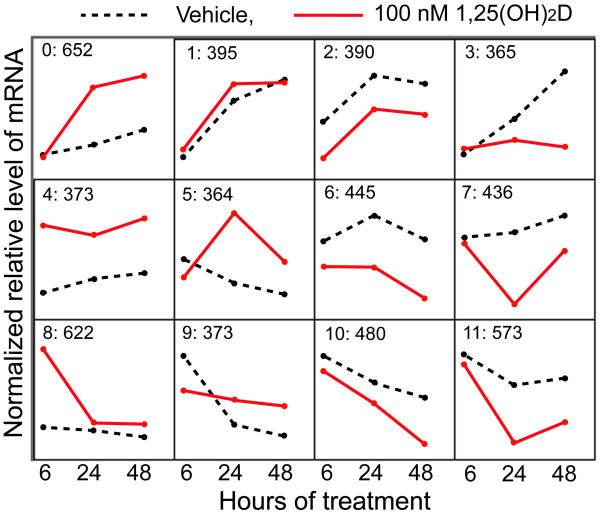
**Self-organizing map analysis of transcripts significantly differentially expressed after 1,25(OH)_2_D treatment in RWPE1 cells**. Transcripts that were found to be significantly differentially expressed (FDR<5%) in at least one time point by 1,25(OH)_2_D treatment (n = 5435) were clustered into 12 groups using self organizing map. The X-axis for each cluster represents hours after treatment (6, 24 and 48 h). The Y-axis for each cluster represents normalized relative level of mRNA expression (in arbitrary units). The number of genes within each cluster in listed at the top, along with the cluster number.

Patterns of 1,25(OH)_2_D-regulated expression were determined using Self Organizing Maps (SOM) (Figure 2). Across the 12 clusters, there were five major expression patterns: Group 1 contained transcripts up-regulated early (clusters 4, 8). Group 2 contained transcripts down-regulated early (clusters 2, 6, 10). Group 3 contained transcripts that were up-regulated at 24 and 48 h (clusters 0, 5). Group 4 contained transcripts that were suppressed at 24 and 48 h (clusters 7 and 11). Group 5 contained transcripts whose normal up-regulation over time was prevented by vitamin D (cluster 3).

### Functional analysis of differentially regulated transcripts

#### Gene Set Analysis (GSA)

Between 6 and 48 h the total number of significantly enriched genesets (curated, motif, and cancer computational) was increased and the proportion of motif and curated genesets that were up-regulated fell (Table 3). In contrast, at 6 h none of the cancer computational genesets were up-regulated but 12 of these genesets were induced at later time points. The lists of the curated, motif, and cancer computational genesets significantly regulated in the GSA analysis are available in Additional files 2, 3 and 4.

**Table 3:** Number of genesets that were significantly altered in GSA analysis of microarray data from 1,25(OH)_2_D treated RWPE1 cells (FDR<5%).

	6 h			24 h			48 h		
Geneset*	c2	c3	c4	c2	c3	c4	c2	c3	c4
**Induced**	69	20	0	49	16	12	47	26	17
**Suppressed**	57	21	16	43	12	20	94	29	25
**Total**	126	41	16	92	28	32	141	55	42

* c2: curated; c3: motif; c4: cancer computational

Genesets that were significantly changed at each time point were grouped according to related function (Table 4). As expected, the motif genesets representing genes containing the classic DR3-type VDRE were significantly enriched by 1,25(OH)_2_D at all three time points. Two other genesets were induced at all three time points: "induced during differentiation" (from c2, curated) and "suppressed by JNK". Genesets suppressed at all time points were for VEGF target genes (i.e. suggesting an anti-angiogenic profile) and cytokine pathways (i.e. an anti-inflammatory profile). Genesets altered by 1,25(OH)_2_D treatment at 6 h only include one induced for "p53 and BRCA1 target genes", suggesting an early pro-apoptosis programming, and suppression of genesets containing WNT-, Notch-, and IGF1- target genes, suggesting the disruption of signals that promote cell proliferation. The shift to a less proliferative, more differentiated cell was supported by the suppression of genesets at 24 and 48 h that contain transcripts whose protein products promote proliferation and suppress differentiation (e.g. "cyclins", "down-regulated during cell cycle arrest").

**Table 4:** Representative genesets identified with GSA analysis as significantly enriched after 1,25(OH)_2_D treatment of RWPE1 cells (FDR<5%).

	Induced	Suppressed
**6 h**	Vitamin D target genes (c3:465, 796, 797) Induced during differentiation (c2:963, 1219,489) Suppressed by JNK (c2:899) Genes involved in electron transport (c2:455) p53 and BRCA1 target genes (c2:1149, 190) GPC Receptors (c2:1108, 613, 620) NRF2 pathway (c2:108) Induced by SERM (c2:528)	VEGF target genes (c2:1600, 1603, 1604, 1605) Cytokines signaling (IL17, STAT3, STEM, LAIR pathways) (c2:406, 853, 861, 861, 1408, 1391) TNFA target genes (c2:1126, 875) Suppressed during differentiation (c2:40) IGF1 target genes (c2:841) WNT target genes (c2:1632, 1634; c3:162) Notch target genes (c2:1087) GPC Receptors (c2:612, 1033, 618) Suppressed by SERM ( c2:528)
		
**24 h**	Vitamin D target genes (c3:465) Induced during differentiation (c2:1219) Suppressed by JNK (c2:899) Genes involved in electron transport (c2:455, 457) Nitrogen metabolism (c2:1096) ROS modulators (c2:758)	VEGF target genes (c2:1600, 1602, 1604, 1605) Cytokines signaling (c2:1650) Down-regulated by p21 (c2:1141, 1138) Cyclins (c2:288) IGF1 target genes (c2:581) Notch signaling (c2:1102) Notch target genes (c2:1087)
		
**48 h**	Vitamin D target genes (c3:465) Induced during differentiation (c2:963) Suppressed by JNK (c2:899) Nitrogen metabolism (c2:1096)	VEGF target genes (c2:1600, 1602, 1604) Cytokines signaling (c2:1100, 1650) IL6 target genes (c2:220, 918, 862) IFNs target genes (c2:1241, 419, 421, 423, 4306, 623, 625, 155, 412, 835, 831, 832, 824, 825, 826, 829, 830) IFNA and G pathway (c2:837, 838, 1394) TNFA target genes (c2:1308, 1476, 1479) Suppressed during differentiation (c2:40, 812) Down-regulated by p21 (c2:1138) IGF1 target genes (c2:581, 841) Induced by JNK (c2:900)STAT2 and STAT5B target genes (c3:597,765)

The geneset collection number shown in parenthesis is followed by ID numbers of changed genesets (c2-curated genesets, c3- motif genesets, see Additional files 2 and 3).

#### GenMAPP and Metacore Analysis

The total number of local maps, GO terms, or Metacore maps influenced by 1,25(OH)_2_D treatment was increased over time, due to an increase in the number of suppressed maps. The detailed results from our GenMapp analysis of local maps and GO terms are available in Additional files 5 and 6, respectively, while the Metacore results are summarized in Additional file 7. A summary of these analyses are provided for local maps and for GO terms in Table 5 and for Metacore in Table 6.

**Table 5:** GenMAPP analysis of the pathways and biological processes modified by 1,25(OH)_2_D treatment in RWPE1 cells.

MAPP Name	%^1^	Z^2^	P^3^	MAPP Name	%	Z	P
**6 h Local maps (Induced): 24 maps at P < 0.1**				**6 h Local maps (Suppressed): 12 maps at P < 0.1**			
Oxidative Stress	35	5.7	0	Wnt Signaling	11	2.2	0.035
MAPK signaling pathway KEGG	13	3.2	0.002	IL-1 NetPath 13	11	1.8	0.076
2-Tissues-Muscle, Fat and Connective	15	2.4	0.031	Phosphatidylinositol signaling system	8	1.8	0.091
				TGFβ signaling pathway	10	1.8	0.092
**6 h GO Biological process (Induced): 236 maps at P < 0.1**				**6 h GO Biological process (Suppressed): 181 maps at P < 0.1**			
Cell differentiation (30154)	5	2.0	0.062	Cell proliferation (8283)	7	3.6	0
Response to stress (6950)	12	3.2	0	Positive regulation of cell proliferation (8284)	8	1.8	0.072
Apoptosis (8219)	30	2.4	0.013	Anti-apoptosis (6916)	10	3.1	0.007
Regulation of transcription from RNA polymerase II promoter (6357)	10	2.5	0.013	Transcriptional repressor activity (16564)	7	2.1	0.039
Signal transduction (7165)	13	7.9	0	Signal transduction (7165)	5	2.8	0.007
Lipid metabolism (6629)	7	2.1	0.035				
**24 h Local maps (Induced): 19 maps at P < 0.1**				**24 h Local maps (Suppressed): 19 maps at P < 0.1**			
Oxidative Stress	23	3.4	0.005	Delta-Notch NetPath 3	15	4.2	0.001
Sterol biosynthesis	28	3.6	0.003	Focal adhesion KEGG	12	4.0	0.001
Nitrogen metabolism	33	4.2	0.005	Hedgehog NetPath 10	27	4.0	0.011
Glycolysis and Gluconeogenesis	15	2.3	0.036	TGF receptor NetPath 7	10	2.8	0.009
2-Tissues-Muscle, Fat and Connective	26	5.4	0	Androgen-Receptor NetPath 2	10	2.4	0.022
**24 h GO Biological process (Induced): 260 maps at P < 0.1**				**24 h GO Biological process (Suppressed): 294 maps at P < 0.1**			
Lipid metabolism (6629)	9	3.1	0.003	Cell proliferation (8283)	8	3.5	0.001
Cell adhesion (7155)	9	2.4	0.014	Cell motility (6928)	11	4.4	0
Development (7275)	7	2.2	0.032	Immune response (6955)	8	4.5	0
Ion transport (6811)	9	2.8	0.01	Signal transduction (7165)	6	5.4	0
**48 h Local maps (Induced): 24 maps at P < 0.1**				**48 h Local maps (Suppressed): 25 maps at P < 0.1**			
Oxidative Stress	23	2.7	0.013	Inflammatory Response Pathway	60	5.4	0
Glutathione metabolism	22	2.2	0.061	Delta-Notch NetPath 3	22	2.3	0.017
Nitrogen metabolism	47	5.4	0	Focal adhesion KEGG	23	3.8	0
Glycerolipid metabolism	18	3.0	0.006	TGF receptor NetPath 7	23	3.7	0
2-Tissues-Muscle, Fat and Connective	28	5.0	0	Androgen-Receptor NetPath 2	23	2.9	0.003
**48 h GO Biological process (Induced): 242 maps at P < 0.1**				**48 h GO Biological process (Suppressed): 245 maps at P < 0.1**			
Lipid metabolism (6629)	12	5.6	0	Cell proliferation (8283)	19	4.3	0
Steroid metabolism (8202)	15	3.8	0	Extracellular matrix (31012)	28	5.7	0
Cell adhesion (7155)	13	3.2	0.001	Immune response (6955)	26	8.5	0
Cell redox homeostasis (45454)	33	2.6	0.033	Angiogenesis (1525)	36	3.8	0
Ion transport (6811)	12	4.2	0	Signal transduction (7165)	14	3.4	0.005

^1^% = percent in map changed; ^2^Z = Z score; ^3^P = permuted P value

**Table 6:** Metacore analysis of the pathways modified by 1,25(OH)_2_D treatment in RWPE1 cells (p < 0.05).

Metacore Map	p-Value	Genes changed
**6 h induced: **83 maps at p < 0.05		
Apoptosis and survival_NGF activation of NF-kB	2.89E-03	6
Transcription: p53 signaling pathway	2.33E-02	5
Inhibitory action of Lipoxins on pro-inflammatory TNF-alpha signaling	2.33E-02	5
Arachidonic acid production	1.76E-03	6
**6 h suppressed: **28 maps at p < 0.05		
Transcription_NF-kB signaling pathway	2.29E-02	4
Development_WNT signaling pathway. Part 2	1.48E-02	6
Development_Notch signaling pathway	4.17E-02	4
Transcription_Androgen receptor nuclear signaling	3.68E-02	5
**24 h induced: **30 maps at p < 0.05		
Oxidative phosphorylation	5.66E-04	13
Cell adhesion_ECM remodeling	2.02E-02	7
Role of tetraspanins in the integrin-mediated cell adhesion	4.62E-02	6
Prostaglandin 2 biosynthesis and metabolism FM	2.93E-03	5
**24 h suppressed: **84 maps at p < 0.05		
Immune response_IL6 signaling pathway	3.85E-03	10
Immune response_IFN gamma signaling pathway	2.06E-04	9
Cell cycle Nucleocytoplasmic transport of CDK/Cyclins	6.04E-05	7
Cell cycle_Role of APC in cell cycle regulation	7.19E-03	6
**48 h induced: **53 maps at p < 0.05		
Cell cycle_Regulation of G1/S transition (part 2)	6.07E-03	6
Oxidative phosphorylation	2.17E-02	11
Regulation of lipid metabolism_Insulin regulation of fatty acid metabolism	1.37E-02	8
Glycolysis and gluconeogenesis (short map)	3.43E-03	7
Apoptosis and survival_p53-dependent apoptosis	3.54E-02	5
Prostaglandin 2 biosynthesis and metabolism FM	1.27E-03	6
**48 h suppressed: **140 maps at p < 0.05		
Cell cycle_Role of APC in cell cycle regulation	2.92E-04	14
Immune response_Antiviral actions of Interferons	4.07E-05	16
Immune response_PGE2 common pathways	5.22E-04	16
Development_TGF-beta receptor signaling	3.94E-04	15
Development_VEGF-family signaling	1.68E-02	9
Peroxisomal branched chain fatty acid oxidation	3.51E-04	8
**All times induced: **66 maps at p < 0.05		
Cytoskeleton remodeling_Keratin filaments	8.37E-04	13
Development_TGF-beta receptor signaling	8.65E-03	13
Development_VEGF signaling and activation	3.92E-02	10
Cell Adhesion_ECM remodeling	3.94E-02	11
Apoptosis and survival_caspase cascade	6.47E-03	9
**All times suppressed: **155 maps at p < 0.05		
Immune Response_IL-27 signaling pathway	3.14E-04	12
Development_Notch Signaling Pathway	5.70E-04	16
Cell cycle_Regulation of G1/S transition (part 1)	6.25E-07	24
Transcription_Androgen Receptor nuclear signaling	3.38E-03	18
Cell adhesion_Ephrins signaling	8.47E-03	14
Development_VEGF-family signaling	3.86E-02	10

An additional GenMAPP analysis was conducted to determine the functional characteristics of each of the five groups from the cluster analysis (see Additional file 8 and 9). Group 1 (up-regulated early) included transcripts for signal transduction, cell differentiation, response to oxidative stress, and lipid metabolism. Group 2 (down-regulated early) contained transcripts for cell proliferation, Wnt and Notch signaling, cell-cell signaling (and cell adhesion), angiogenesis, and the immune/inflammatory response. Group 3 (up-regulated late) included transcripts for cellular metabolism, transition metal binding, and cell redox homeostasis. Group 4 (down-regulated late) contains the largest number of functional groups related to cell proliferation and also contained maps for the EGFR signaling pathway and sphingolipid metabolism. Group 5 (prevention of up-regulation) reflects transcripts regulating a defense/inflammatory response and anti-apoptotic signaling.

We also looked at the functional categories that were regulated by 1,25(OH)_2_D treatment at each time point. The most prominent functional groups/maps induced at 6 h were for cell differentiation, apoptosis, lipid metabolism, and markers of the response to oxidative stress. The GO process for lipid metabolism, the local map for tissue-muscle, fat, and connective (which contains general markers of cell differentiation), and maps for oxidative stress (i.e. the local map for oxidative stress and glutathione metabolism, the GO process of cell redox homeostasis) were induced at 6 h and at later time points. Similarly, Metacore maps related to apoptosis, keratins, and "cell adhesion through extracellular modeling" were up-regulated when all time points were evaluated together.

The primary suppressed function that is consistent with a hypothesized role for vitamin D in cancer prevention was cell proliferation. At the later time points a clear reduction in specific functional groups and maps related to "cell cycle regulation" was observed. Consistent with this, the local map and Metacore maps for Wnt signaling were suppressed at 6 h and in the Metacore analysis, Notch signaling maps were significantly suppressed at 6 h and for all time points combined. Several other interesting processes that were down-regulated significantly at later time points include: angiogenesis (i.e. VEGF family signaling), androgen receptor signaling, and various aspects of the cytokine signaling (e.g. in Metacore this was reflected at 24 h as IL-6 and IFN signaling as well as in the combined timepoint analysis as IL-1 and IL-27 signaling).

## Discussion

Optimal vitamin D status been proposed to prevent prostate carcinogenesis [3] and this anticancer activity is most likely mediated through VDR-dependent changes in the prostate transcriptome [4]. By applying microarray technology to the immortalized but non-tumorigenic human prostate epithelial cell line RWPE1, we have identified a number of mechanisms by which vitamin D may influence the early stages of prostate carcinogenesis. Our data show that 1,25(OH)_2_D influences many pathways relevant to prostate carcinogenesis and they underscore the critical role of this molecule in the maintenance of prostate epithelial development, function, and turnover.

Several studies have shown that 1,25(OH)_2_D treatment causes cell cycle arrest and growth suppression of primary prostate epithelial cell lines or prostate cancer cell lines. While this has been viewed as the major anticancer effect for 1,25(OH)_2_D, the mechanism accounting for this effect is not known with certainty. Liu et al. showed that the cyclin-dependent kinase (CDK) inhibitor p21 was strongly induced by 1,25(OH)_2_D treatment in the monocytic cell line HL-60 and they identified a functional Vitamin D Response Element (VDRE) in the p21 promoter [9]. However, 1,25(OH)_2_D does not increase p21 transcript level in LNCaP cells [18] and our data show that the impact of 1,25(OH)_2_D on p21 mRNA level is modest (1.32-fold at 6 h). Another CDK inhibitor, Wee1, is induced by 1,25(OH)_2_D in cultured keratinocytes leading to G2/M arrest [23] and this is also modestly induced by 1,25(OH)_2_D in RWPE1 cells (1.4-fold at 6 h). We examined other transcripts related to cell cycle control in our study but most of these were suppressed only at the later time points: e.g. GAS6, ETS1, CDK6, cyclin B2, cyclin A, CDC25C, and CDC27. This suggests that they are not primary effects of 1,25(OH)_2_D action.

In contrast, our microarray data suggest that disruption of Wnt-signaling may be an alternative mechanism for 1,25(OH)_2_D-mediated growth arrest. A number of Wnt pathways and genesets were reduced by 1,25(OH)_2_D treatment by 6 h, e.g. the geneset containing genes with LEF1/TCF4 binding motifs in their promoters that includes classical Wnt target genes like c-myc, cyclin D, PPARδ (see tables 4, 5 and 6). This is consistent with a model developed for colonocytes where VDR directly interacts with β-catenin to disrupt transcriptional events that normally increase cell proliferation [24,25]. 1,25(OH)_2_D treatment also induced E-cadherin mRNA 1.7-fold in RWPE1 cells at 6 h. E-cadherin antagonizes Wnt signaling by inducing translocation of β-catenin to the plasma membrane (see figure in Additional file 10 for a summary of the transcript-level changes occurring in Wnt signaling). Disruption of Wnt/β catenin signaling could be a means whereby vitamin D treatment amplifies its impact on biology. In support of this model, network analysis in Metacore identified a gene network with the β catenin/TCF gene target c-myc at its center. This network connects the suppression of c-myc expression to a large number of other transcripts that were differentially expressed by 1,25(OH)_2_D treatment (see Additional file 11). However, while our data shows a consistent, early suppression of Wnt/β-catenin signaling, careful experimental evaluation of this hypothesis in prostate epithelial cells is needed.

Our data also show that transcripts for the Notch ligands, JAG1, JAG2, and DLL1 were suppressed by 1,25(OH)_2_D treatment in RWPE1 cells (each reduced by -2 fold at 6 or 24 h). NOTCH1 and JAG1 are proposed as markers of normal prostate stem cells and they are necessary for fate determination of the proliferating stem cell [26]. In addition, expression of JAG1 protein is increased in metastatic prostate cancer [27] and prostate cancer cells suggesting Notch1 and JAG1 mediated signaling may enhance carcinogenesis [26,28]. Collectively, these observations suggest that suppression of Notch or its ligands by 1,25(OH)_2_D could be associated with cancer protection.

In addition to modulating cell grown, vitamin D has been proposed to inhibit the development of the tumor vasculature that is required for the progression of solid tumors [29-32] by suppressing expression of Vascular Endothelial Growth Factor (VEGF) family members, the major pro-angiogenic cytokines in normal prostate epithelial cells [33]. The impact of 1,25(OH)_2_D on VEGF gene regulation has been confusing. In mouse embryo fibroblasts and human vascular smooth muscle cells 1,25(OH)_2_D induces VEGFA expression through a VDRE in its promoter [34], yet 1,25(OH)_2_D can also suppress VEGF-induced vasculogenesis in cultured endothelial cells and in nude mice implanted with MCF-7 breast cancer cells [35]. We found that 1,25(OH)_2_D treatment suppressed VEGFC mRNA levels at all time points (-2.1 to -1.6 fold). Higher expression of VEGFC occurs after NKX3.1 loss in prostate cancer and is correlated with lymph node metastasis of prostate cancer [36]. VEGF promotes angiogenesis by binding to and activating the receptors KDR, FLT1, and NRP1 and we found that 1,25(OH)_2_D significantly suppressed KDR and NRP1 expression. Suppressing the activation of these receptors reduces tumor angiogenesis and promotion in the Dunning cell carcinoma model [37]. Finally, VEGF signaling can be suppressed by competitive binding of semaphorins to NRP1. Semaphorins induce apoptosis, inhibit growth of lung and breast tumor cells [38], and modulate invasion and adhesion of prostate cancer cells [39]. In our study, 1,25(OH)_2_D induced expression of several semaphorin isoforms including SEMA3B, 3F, and 6D (19.4-, 2.5-, and 18-fold, respectively at 6 h). Collectively our array data show that 1,25(OH)_2_D induces an anti-angiogenic transcript profile in RWPE1 cells.

While our discussion has focused on the modulation of classical anti-cancer effects, our array analysis also revealed other potential mechanisms for vitamin D mediated cancer prevention. For example, oxidative stress-induced damage of DNA and other cellular components are implicated in cancer [40]. These effects can be prevented by induction of antioxidant defense or DNA repair mechanisms that subsequently reduce the biological impact of reactive oxygen species. In our study 1,25(OH)_2_D influenced the expression of genes related to these events (see figure in Additional file 12) and our observations are consistent with a previous microarray study in SCC25 cells that showed EB1089-regulated induction of transcripts whose gene products are involved in antioxidant (e.g. thioredoxin reductase 1, TXNRD1) and DNA repair processes (e.g. GADD45α) [16]. There is some evidence that 1,25(OH)_2_D directly regulates transcripts controlling these functions. Glucose-6-phosphate dehydrogenase (G6PD) is an enzyme involved in maintaining cellular glutathione levels and its mRNA was significantly induced at all time points following 1,25(OH)_2_D treatment in RWPE1 cells (3.4-6.8 fold). Recently, Bao et al. [41] showed that G6PD expression is controlled by 1,25(OH)_2_D in prostate epithelial cells through a VDRE located in the first intron of the gene and that the induction of G6PD by 1,25(OH)_2_D protected RWPE1 cells against H_2_O_2_-induced apoptosis. It is also possible that vitamin D-mediated protection from pro-oxidant stress is indirect due to the induction of nuclear factor (erythroid-derived 2)-like 2 (NFE2L2), a transcription factor that controls expression of genes for many antioxidant enzyme systems [42]. NFE2L2 expression is down-regulated in prostate cancer and suppression of NFE2L2 promotes prostate tumor development in TRAMP mice [43]. Consistent with a role for NFE2L2 in vitamin D-mediated cancer prevention, a number of NFE2L2 target genes were increased in RWPE1 cells after 1,25(OH)_2_D treatment, e.g. GPX3, HMOX1, AKR1C2, and TXNRD1.

Several studies have shown that vitamin D is anti-inflammatory and our data are consistent with these findings. In the immune system 1,25(OH)_2_D promotes immunotolerance and immunosuppression by altering the differentiation and function of tolerogenic dendritic cells [44], suppressing NFkB signaling necessary for T helper cell activation [45], and increasing the activity of regulatory T cells necessary for immunosuppression [46]. These actions would be expected to protect tissues from pro-inflammatory stresses that cause prostatitis [47] and promote prostate carcinogenesis [48]. However, many cells outside of the traditional immune system have the capacity to respond to and produce immuno-modulatory factors and we found that in RWPE1 cells vitamin D-treatment regulated a large number of transcripts for proteins controlling immune function. In fact, induction of CD14 was one of the most strongly 1,25(OH)_2_D up-regulated transcripts in RWPE1 cells. Prostate epithelial cells are thought to be early sensors of infection and CD14 and toll-like receptor 4 (TLR4) production in these cells contributes to protection from Chlamydia infection [49]. While a role for vitamin D-induced CD14 or TLR4 induction in the regulation of prostate infection/inflammation has not been studied directly, others have identified CD14 as crucial factor for vitamin D induced expression of the antimicrobial peptide cathelicidin in human keratinocytes [50].

Another point where vitamin D may inhibit inflammatory processes is through suppression of cytokine signaling and production. Consistent with this, we found that 1,25(OH)_2_D suppressed several components of JAK-STAT signaling in RWPE1 cells including JAK1, STAT1, n-myc and STAT interactor (NMI), STAT2, and STAT3. JAK-STAT signaling is required for the pro-proliferative effects of many cytokines including the pro-inflammatory cytokines IL6, IL12 and IFNγ [51]. In addition, transcripts for many cytokine receptor transcripts (i.e. upstream regulators of JAK-STAT signaling) and cytokines (i.e. downstream targets of JAK-STAT signaling) were suppressed by 1,25(OH)_2_D-treatment (see figure in Additional file 13). Disruption of JAK-STAT signaling could be a means whereby vitamin D treatment amplifies its impact on the prostate epithelial cells. In support of this model, network analysis of the data from the 48 h timepoint identified a gene network with STAT1, STAT3, and the transcription factor PU.1 at three interacting centers. This network connects the suppression of STAT1 and STAT3 expression to a large number of other transcripts related to immunoregulation that were differentially expressed by 1,25(OH)_2_D treatment (see Additional file 14). Many changes in immune or cytokine signaling pathways occur only at or after 24 h of treatment but this reflects a clear anti-inflammatory role for 1,25(OH)_2_D in prostate epithelial cells that is consistent with findings by others in Jurkat T cells [52] and Th1 immune cells [53]. In addition, Nonn et al. had previously shown that in normal prostate epithelial cells, 1,25(OH)_2_D inhibits TNFα-induced IL-6 production through a mechanism that requires direct transcriptional regulation of the MAPK phosphatase 5 gene (DUSP-10, increased 9.9-fold at 6 h in our analysis) [54]. Finally, our data suggest that NFkB signaling is modulated by 1,25(OH)_2_D treatment; i.e. upregulation of IkB (NFKBIA) expression and suppression of RELB mRNA levels. These observations are consistent with data showing that 1,25(OH)_2_D suppressed secretion of IL-8 by interfering with NFκB signaling in RWPE1 cells [31] and that it enhanced radiosensitivity of prostate cancer cells by selectively suppressing radiation-mediated RELB activation in prostate cancer cell lines [55].

Prostaglandin signaling is a final pro-inflammatory pathway that has been identified as vitamin D regulated by others. Krishnan et al. [18] found that the message for the prostaglandin inactivating enzyme 15-PGDH was significantly increased and the mRNA levels for COX2, an enzyme that drives production of PGE2 levels, was significantly reduced by 1,25(OH)_2_D in LNCaP cells. Moreno et al. [56] subsequently showed that 1,25(OH)_2_D reduced the mRNA levels for two prostaglandin receptors (EP2, FP) in prostate cancer cells. Since the prostanoid pathway is a critical component in acute inflammation that may contribute to the development of prostate cancer [57], this suggested that vitamin D-mediated chemoprevention involves disruption of prostaglandin signaling. However, our RWPE1 data is not consistent with this hypothesis. In contrast to the earlier studies in LNCaP cells, we observed induction of COX2 by 1,25(OH)_2_D in RWPE1 cells (6.3-fold at 6 h) and neither 15-PGDH nor prostaglandin receptor mRNA levels were altered.

The functional analysis of transcript-levels changes induced by 1,25(OH)_2_D reveals how the biology of prostate epithelial cells is changed by the hormone but microarray studies cannot differentiate between transcripts that are differentially regulated due to direct, VDR-mediated transcriptional activation and those that are secondary effects following the primary transcriptional events. As such, we can only infer the direct VDR gene targets based upon our data and their relationship to other studies. Unfortunately, there is very little information regarding the effect of 1,25(OH)_2_D on the prostate epithelial cell transcriptome to draw upon from earlier studies. Using a spotted cDNA microarray with no sample replicates, Peehl et al. [18,22] identified 48 transcripts as 1,25(OH)_2_D regulated in primary normal prostate epithelial cells and 52 transcripts in primary cultures of prostate cancer cells. Twenty-one of the differentially regulated transcripts from the normal prostate epithelial cells and 28 of the transcripts from the primary prostate cancer cells were also differentially regulated by 1,25(OH)_2_D in RWPE1 cells. Still, the only overlap between the three lists was CYP24, DUSP10, AKAP12, P2RY2, BMP6, TGFB2, and TXNRD1. In contrast, of the 22 transcripts identified by Krishnan et al. [18,22] as differentially regulated in 1,25(OH)_2_D-treated LNCaP cells only IGFBP3, ABCA1 and FKBP5 were regulated in the same direction in RWPE1 cells. This suggests that the response of RWPE1 cells is more similar to that of primary cultures of human prostate epithelial cells.

Of the genes identified in the three different prostate array studies only CYP24 has been identified as a direct target for 1,25(OH)_2_D. Our ChIP examination of the AKAP12 and CYP26B1 promoters revealed significant VDR binding to putative VDREs in those promoters too, but given the large number of transcript level changes we observed, we expect that many more primary VDR target genes exist in the prostate epithelial cells. Before our analysis, the most comprehensive array-based analysis of 1,25(OH)_2_D action and putative VDR target genes was conducted by Wang et al. [17]. Using a bioinformatics approach they identified putative VDREs in the promoters of genes whose transcripts were differentially regulated by treatment with EB1089 in SSC25 cells (12 h in presence of cycloheximide). We compared their list of differentially expressed transcripts to our list of transcripts regulated by 1,25(OH)_2_D at 6 h in RWPE1 cells based on the assumption that the early time point is less likely to contain transcripts that are regulated as a secondary consequence of primary vitamin D-mediated transcription events. This analysis identified 414 transcripts that were vitamin D regulated in both cell lines. 267 of these had a putative VDRE (see Additional file 15), including 16 of the 21 transcripts regulated by 1,25(OH)_2_D in both RWPE1 and primary prostate epithelial cells [22]. This suggests there may be a much larger number of direct VDR target genes than has been suggested by earlier research. Future studies using either ChIP-chip or ChIP-sequencing [58] will be necessary to validate whether these 267 transcripts are truly direct vitamin D target genes.

## Conclusions

This study is the most comprehensive functional analysis of 1,25(OH)_2_D-induced changes in the transcript profile of non-tumorigenic prostate epithelial cells. As such, it provides new insight into the mechanisms used by 1,25(OH)_2_D to prevent the early stages of prostate cancer. By using several independent procedures we identified multiple 1,25(OH)_2_D-regulated pathways and mechanisms that may disrupt the promotion of carcinogenesis *in vivo*. This includes anticancer mechanisms that have been traditionally attributed to 1,25(OH)_2_D action, e.g. suppression of cell proliferation and angiogenesis, as well as several potential new mechanisms including gene-protective and immunosuppressive effects. Further research is necessary to determine whether the genes identified in our array study are direct VDR target genes as well as to determine whether these transcripts are regulated by vitamin D signaling *in vivo*.

## Methods

### Supplies

Unless otherwise noted, all chemicals were obtained from Sigma (St. Louis, MO). Defined Keratinocyte-Serum Free medium (SFM) and RPMI medium 1640 were obtained from Invitrogen (Carlsbad, CA) and cell culture plasticware was from Corning-Costar (Cambridge, MA). 1,25(OH)_2_D was purchased from Biomol International (Plymouth Meeting, PA).

### Cell culture

RWPE1 cells [59] were obtained from ATCC (CRL-11609) (Manassas, VA) at passage 52 and used between passages 55 and 60. Cells were maintained in Defined Keratinocyte-SFM medium supplemented with growth factors (insulin, Epidermal Growth Factor and Fibroblast Growth Factor, Invitrogen, Carlsbad, CA) and medium was replaced every the other day. LNCaP cells were obtained from ATCC (CRL-1740D) and used between passages 25 and 30. LNCaP cells were grown in RPMI-1640 medium supplemented with 10% fetal bovine serum and antibiotics.

### Microarray analysis

#### Cell Treatment

RWPE1 cells were plated in T75 flasks (1 × 10^6 ^cells per flask) and grown until cells reached 60% confluence. At this point, cells were treated with medium containing 100 nM of 1,25(OH)_2_D or vehicle (0.1% ethanol) for 6, 24 or 48 h (n = 4 per treatment, 24 total samples). For the 48 h time point, media was replaced at 24 h prior to cell harvest. Total RNA was isolated from the cells using TriReagent (Molecular Research Center, Inc., Cincinnati, OH) in accordance with the manufacturer's instructions. Isolated total RNA was further purified using the RNeasy kit (Qiagen, Valencia, CA). The quality of the isolated RNA was confirmed using agarose gel electrophoresis.

#### Microarray Data Analysis

The transcripts levels in each sample were determined by using the Affymetrix HU133 plus 2.0 GeneChip (Affymetrix, Santa Clara, CA; 54,210 probe sets covering over 47,000 transcripts and splice variants). RNA labeling, chip hybridization and chip scanning was carried out at the Ohio State University Comprehensive Cancer Center by (Columbus, OH) using standard Affymetrix protocols (Affymetrix, Santa Clara, CA). Chips were scanned and raw data was saved into CEL files or analyzed using the Affymetrix Microarray Suite (MAS) 5.0 software. Microarray data may be accessed at the NCBI Gene Expression Omnibus (GEO) database (accession # GSE15947).

#### Quality Control Assessment, Chip Normalization, and Filtering

The quality of microarray images from individual chips was examined using methods from the affyPLM package of Bioconductor http://www.Bioconductor.org[60]. The quality of the expression distribution at the probeset level between chips was inspected by using the Relative Log Expression (RLE) and the Normalized and the Unscaled Standard Error (NUSE) methods. All of the chips were found to be of high quality and were used for subsequent analysis.

Microarray data from CEL files for all 24 chips was normalized simultaneously and expression levels were generated using the gcRMA package in Bioconductor [61]. The normalized data was filtered for present/absent call using information from MAS5.0 software ("Present", P < 0.05; "Marginal", 0.05 < P ≤ 0.065, "Absent", P > 0.065). Only transcripts identified by MAS5 as "present" or "marginal" in 3 out of 4 replicates for at least one treatment group were retained for further analysis. Of the 54,210 transcripts represented on the chip, 25,986 met our present/absent filter criterion.

#### Statistical Analysis of Microarray Data

Differentially expressed transcripts were identified at each time point (control vs. 1,25(OH)_2_D treated) using the Significance Analysis of Microarray program (SAM, version 3.02) [62]. Data for each time point were analyzed using a two class, unpaired analysis with T-statistics and 100 permutations. For each transcript SAM uses permutation of the data to identify a False Detection Rate (FDR) that balances type I and type II statistical error rates [63]. Significance for differential expression due to 1,25(OH)_2_D treatment was determined at the 5% FDR at each time point.

#### Clustering of Microarray Data

Patterns of differentially expressed genes were determined by cluster analysis using Self-Organizing Maps in GeneCluster http://www.genome.wi.mit.edu/MPR/. Only data from transcripts identified by SAM as significantly differentially expressed in at least one time point (FDR<5%), were used for the cluster analysis. Prior to the clustering, the data was normalized to mean = 0 and variance = 1. For this analysis we used a 4 × 3 matrix and the default settings of the software (random vectors method of initialization, bubble neighborhood definition, α_i _= 0.1, σ_i _= 5, α_f _= 0.005, σ_f _= 0.5).

#### Functional Analysis of Microarray Data

Gene Set Analysis (GSA) was conducted using the GSA function in SAM http://www-stat.stanford.edu/~tibs/GSA/ and geneset databases from the Molecular Signatures Database http://www.broad.mit.edu/gsea/msigdb/index.jsp: c2-curated, c3-motif, and c4-cancer neighborhood. Analysis for each time point was done using a two class, unpaired analysis with T-statistics, 1000 permutations, automatic estimation of s0 factor for denomination, K-nearest Neighbors imputer and a random number seed.

Two methods were used to identify and visualize biological processes and pathways that were enriched due to 1,25(OH)_2_D treatment: GeneMAPP http://www.genmapp.org[64] and Metacore software (Metacore, St. Joseph, MI). GenMAPP analysis was done for each of the time points as well as on groups of related clusters from the cluster analysis (group 1 (clusters 4, 8), group 2 (clusters 2, 6, 10), group 3 (clusters 0, 5), group 4 (clusters 7, 11), group 5 (cluster 3)). Criteria for including a transcript into the GeneMAPP analysis was FDR <5% and fold change >1.2 or < -1.2. The search for maps significantly enriched in the transcripts that meet this criterion was done separately for up-regulated and suppressed genes using the Gene Database (Hs-Std_20060526), local maps (Hs_Contributed_20070308, Hs_KEGG_Converted_20041111 and Hs_Tissue-specific_20050711) and gene ontology (GO) maps (Hs_GO_Samples_20050810). GeneMAPP maps with p-value <0.1 were considered significant.

For the Metacore analysis, transcripts significantly differentially induced or suppressed (SAM, FDR<5% ) were mapped to Metacore maps representing functional pathways individually for each time point or for all time points combined. Enrichment of Metacore maps was determined by the p-value of hypergeometric distribution representing probability that a transcript would be matched to a map by chance. This analysis takes into account the sizes of the dataset, map or ontology group. Metacore maps with p-value < 0.05 were considered significant. Networks were generated de novo from the differentially expressed transcripts at each time point by using Metacore. In addition, the "Analyze network (transcription factors)" program was used to create networks centered around specific transcription factors of interest.

### Validation of microarray results

For the validation of microarray data, differential expression of 11 genes was examined in cDNA prepared from the RNA for the microarray study by Real Time Polymerase Chain Reaction (RT-PCR). The impact of 1,25(OH)_2_D treatment on the accumulation of these 11 transcripts was also examined in RNA from 1,25(OH)_2_D-treated LNCaP cells and in primary normal human prostate epithelial cells. 80% confluent LNCaP cells were treated with medium containing 100 nM of 1,25(OH)_2_D or vehicle (0.01% ethanol) for 6 h. RNA from 1,25(OH)_2_D-treated primary human prostate epithelial cells was a provided by Dr. Scott Cramer (Wake Forest University, Winston-Salem, NC). Three human primary prostate epithelial cell preparations were treated with growth medium containing 10 nM of 1,25(OH)_2_D or vehicle (0.01% ethanol) for 8 h. Cells were harvested in TriReagent and RNA isolated. cDNA was prepared from RNA samples as we have described previously [65].

cDNA samples were analyzed by RT-PCR analysis using the BioRad MyiQ Real-Time PCR system and the BioRad SYBR Green supermix (BioRad Laboratories, Hercules, CA). Expression levels were determined from the threshold cycle (Ct) value using the method of 2^-ΔΔCt ^described elsewhere [66] and using GAPDH expression as the reference control gene. Primers sequences for RT-PCR are listed in Additional file 16. The cycle conditions for the PCR were 1 cycle of 3 minutes at 95°C and 40 cycles of 30 seconds at 95°C, 30 seconds at the annealing temperature, and 30 seconds at 72°C.

### Identification of putative vitamin D receptor binding sites in selected genes

The promoters of the genes encoding the top 50 transcripts significantly up-regulated by 1,25(OH)_2_D at 6 h, were analyzed for presence of VDR binding sites using a bioinformatics approach. The -10 kb to +10 kb region (transcriptional start site = 0) of candidate genes was downloaded from the GenBank database at the NCBI. We then screened the conserved regions of the promoters utilizing an *in silico *approach by using CONSITE http://asp.ii.uib.no:8090/cgi-bin/CONSITE/consite/ and the following settings: window size = 50 bp; conservation cut-off = 70%, Transcription Factor score threshold = 65%. Putative VDR binding sites with similarity score over 3.5 were accepted as candidates for ChIP validation. Ten potential VDR binding sites located within conserved regions were selected for further testing within the following gene promoters: SEMA3B, CD14, P2RY2, AKAP12, SERPINB1, HBEGF, TXNRD1, CYP26B1, MTSS1 and LOX. The location of these sites is provided in Additional file 15.

### Chromatin Immunoprecipitation (ChIP) assays

RWPE1 cells were cultured to 60% confluence and treated with 10 nM 1,25(OH)_2_D or vehicle for 3 h. ChIP assays for VDR association to DNA were done as we have previously described [67]. ChIP assays were performed with anti-VDR antibody sc-1008 from Santa Cruz Biotechnology, Inc., (Santa Cruz, CA). The primers used for VDRE region in CYP24 promoter (-300 bp from transcription start site (TSS), TRPV6 promoter (-4.3 kb to TSS) and SEMA3B promoter (+2 kb to TSS) were described previously [17,68]. For other target genes, primer pairs were designed with PRIMER3 http://frodo.wi.mit.edu to amplify a fragment containing the predicted VDR binding sites. Primers used for analysis of CD14, P2RY2, AKAP12, SERPINB, HBEGF, TXNRD1, CYP26B1, MTSS1 and LOX are provided in Additional file 17. GAPDH primers used as positive controls for the ChIP assay were obtained from Upstate Biotechnology (Lake Placid, NY).

### Statistical analysis for non-array experiments

The analysis for treatment effects in the RT-PCR data was conducted using one-way ANOVA using the SAS statistical software package (SAS 8.0 Cary, NC). Pairwise comparisons were conducted when appropriate using Fisher's Protected LSD. Values are expressed as the means ± SEM. Differences between means were considered significant at p < 0.05.

## Abbreviations

15-PGDH: hydroxyprostaglandin dehydrogenase 15-(NAD); AKAP12: a kinase (prka) anchor protein 12; AKR1C2: aldo-keto reductase family 1, member C1; APCDD1: adenomatosis polyposis coli down-regulated 1; BMP6: bone morphogenetic protein 6; BRCA1: breast cancer 1; CALML3: calmodulin-like 3; CD14: CD14 antigen; CDC25C: cell division cycle 25 homolog C (S. pombe); CDC27: cell division cycle 27 homolog (S. cerevisiae); CDK: cyclin-dependent kinase; CDK6: cyclin-dependent kinase 6; ChIP: chromatin immunoprecipitation; COX2: prostaglandin-endoperoxide synthase 2; CYP24: cytochrome p450, family 24, subfamily A, polypeptide 1; CYP26B1: cytochrome p450, family 26, subfamily b, polypeptide 1; DLL1: delta-like 1 (Drosophila); DR3: direct repeat with 3 bp spacing; DUSP10: dual specificity phosphatase 10; EGFR: epidermal growth factor receptor; ETS1: v-ets erythroblastosis virus E26 oncogene homolog 1 (avian); FDR: false discovery rate; FLT1: fms-related tyrosine kinase 1; G6PD: glucose-6-phosphate dehydrogenase; GADD45a: growth arrest and DNA-damage-inducible, alpha; GAPDH: glyceraldehyde-3-phosphate dehydrogenase; GAS6: growth arrest specific 6; GCRMA: GC Robust Multi-array Average; GO: gene ontology; GPX3: glutathione peroxidase 3; GSA: gene set analysis; HBEGF: heparin-binding egf-like growth factor; HMOX1: heme oxygenase (decycling) 1; IGF1: insulin-like growth factor 1; IGFBP3: insulin-like growth factor binding protein 3; IL-17: interleukin 17; IL-27: interleukin 27; IL-6: interleukin 6; JAG1: jagged 1; JAG2: jagged 2; KDR: kinase insert domain receptor; LEF1: lymphoid enhancer binding factor 1; LOX: lysyl oxidase; MTSS1: metastasis suppressor 1; NFE2L2: nuclear factor (erythroid-derived 2)-like 2; NFKBIA: nuclear factor of kappa light polypeptide gene enhancer in B-cells inhibitor, alpha; NOTCH1: Notch homolog 1, translocation-associated (Drosophila); NRP1: neuropilin 1; NUSE: normalized and the unscaled standard error; P2RY2: purinergic receptor p2y, G-protein coupled 2; PCR: polymerase chain rreaction; PPARδ: peroxisome proliferator-activated receptor delta; RAD9A: RAD9 homolog A (S. pombe); RELB: v-rel reticuloendotheliosis viral oncogene homolog B; RLE: relative log expression; ROS: reactive oxygen species; RT-PCR: real time polymerase chain reaction; SAM: significance analysis of microarray; SEM: standard error of the mean; SEMA3B: semaphorin 3B; SEMA3F: semaphorin 3F; SERPINB1: serpin peptidase inhibitor, clade b (ovalbumin), member 1; SEM: standard error of the mean; SFM: serum free medium; SULT1A3: sulfotransferase family, cytosolic, 1A, phenol-preferring, member 3; TCF4: transcription factor 4; TGFβ1: transforming growth factor, beta 1; TLR4: toll-like receptor 4; TSS: transcription start site; TXNRD1: thioredoxin reductase 1; VAV3: vav 3 oncogene; VDR: vitamin D receptor; VDRE: vitamin D response element; VEGF: vascular endothelial growth factor; VEGFA: vascular endothelial growth factor A; VEGFC: vascular endothelial growth factor C; WNT: Wingless-type MMTV integration site family.

## Competing interests

The authors declare that they have no competing interests.

## Authors' contributions

PLK conducted the RWPE1 experiment, performed statistical, functional and cluster analysis of microarray data, conducted validation of target genes using QRT-PCR and prepared the manuscript. ZZ carried out preliminary studies and participated in the RWPE1 experiment and sample preparation. MC conducted ChIP experiments and bioinformatic identification of VDREs in the promoter regions of genes. SKC was responsible for microarray analysis and provided interpretation of the data. JCF was responsible for the overall design of the project, data interpretation and final paper editing. All authors of the paper read and contributed to the final manuscript.

## Supplementary Material

Additional file 1 **Significantly differentially expressed transcripts**. Significantly changed transcripts at any time point from SAM analyzed microarray data on RWPE1 cells treated 100 nm 1,25(OH)_2_D vs. vehicle. Click here for file

Additional file 2 **GSA currated gene sets c2**. GSA curated genesets (c2) significantly enriched in differentially expressed transcripts. Click here for file

Additional file 3 **GSA motif genesets c3**. GSA motif genesets (c3) significantly enriched in differentially expressed transcripts. Click here for file

Additional file 4 **GSA cancer computational genesets (c4)**. GSA cancer computational genesets (c4) significantly enriched in differentially expressed transcripts. Click here for file

Additional file 5 **GenMAPP local map results**. Table of GenMAPP analysis of local maps for enrichment (SAM, FDR<5%). Click here for file

Additional file 6 **GenMAPP GO group results**. GenMAPP Gene Ontology terms significantly changed (Z > 0, Permute P <= 0.1). Click here for file

Additional file 7 **Metacore summary**. Metacore Maps enriched in significantly changed genes (SAM, <5%FDR). Click here for file

Additional file 8 **GeneMAPP SOM groups Local maps results**. Table showing GenMAPP analysis on SOM groups using Local maps. Click here for file

Additional file 9 **GeneMAPP SOM groups GO group results**. Table of GeneMAPP GO map analysis for SOM groups. Click here for file

Additional file 10 **Effect of 1,25(OH)_2_D on Wnt and Notch signaling at 6 h**. Figure showing the effect of 1,25(OH)_2_D (100 nM, 6 h) on transcripts controlling Wnt and Notch signaling in RWPE1 cells. Differentially expressed transcripts (SAM, any time point, FDR<5%) were examined by time point for functional changes using GenMAPP and GSA. The GeneMapp local map for Wnt signaling (Hs_WNT_Signaling) was identified as significantly down regulated at 6 h. In addition, a GSA motif geneset (c3 #162) for genes containing Lef1 domains in their promoters (a Wnt pathway targeted transcription factor) was significantly down-regulated. Click here for file

Additional file 11 **Effect of 1,25(OH)_2_D on c-Myc transcriptional activity at 6 h**. Image representing effect of vitamin D induced supression of c-Myc on the mRNA level of c-myc target genes. Significantly differentially expressed transcripts at 6 h (SAM, FDR<5%) were analyzed by using Metacore Network analysis (Transcription factor). Up--regulated genes are marked with red circles; down--regulated with blue circles. Arrows are color coded to reflect the known regulatory action between two proteins. Red arrows between proteins indicates a negative regulatory effect, green arrows indicate a positive regulator effect, gray arrows indicate an unspecified regulatory effect. Click here for file

Additional file 12 **Effect of 1,25(OH)_2_D on antioxidant and DNA protection at 6 h**. Figure showing the effect of 1,25(OH)_2_D (100 nM, 6 h) on transcripts controlling antioxidant and DNA repair systems in RWPE1 cells. Differentially expressed transcripts (SAM, any time point, FDR<5%) were examined by time point for functional changes using GenMAPP and GSA. The GenMAPP local map for antioxidant responses to reactive oxygen (Hs_Oxidative_Stress) was identified as significantly up-regulated at 6 h. While not on this map, the up-regulation of G6PD is also relevant as this enzyme system contributes to glutathione production. Click here for file

Additional file 13 **1,25(OH)_2_D suppresses proinflammatory cytokine signaling**. Figure representing regulation of transcripts controlling cytokine signaling in RWPE1 cells by 1,25(OH)_2_D treatment (100 nM). Differentially expressed transcripts (SAM, any time point, FDR<5%) were examined for functional changes using GenMAPP and GSA. A large number of pathways related to the signaling through cytokine pathways were identified as down-regulated. Most of these pathways utilize a JAK-STAT intracellular signaling pathway. A selection of transcripts affected and their relationship to JAK-STAT signaling are shown. Click here for file

Additional file 14 **1,25(OH)_2_D suppresses STAT1, STAT3 and PU.1 networks at 48 h**. Image representing suppression of transcripts regulated by STAT1, STAT3 and PU.1. Significantly differentially expressed transcripts at 48 h (SAM, FDR<5%) were analyzed by using Metacore Network analysis (Transcription factor). Most of these transcripts are regulated by STAT1 or STAT3. Up--regulated transcripts are marked with red circles; down--regulated transcripts are identified by blue circles. Arrows are color coded to reflect the known regulatory action between two proteins. Red arrows between proteins indicates a negative regulatory effect, green arrows indicate a positive regulator effect, gray arrows indicate an unspecified regulatory effect. Click here for file

Additional file 15 **VDRE containing genes, comparison with Wang et al, 2005**. Comparison of VD regulated transcripts in RWPE1 to those reported for EB1089 by Wang et al. in SCC25 cells. Click here for file

Additional file 16 **RT-PCR primers**. List of RT-PCR primers used for validation of differential expression identified in 1,25(OH)_2_D-treated RWPE1 cells. Click here for file

Additional file 17 **Genes tested for functional VDRE**. Table of genes tested for functional VDRE in promoter regions. Click here for file
