# Supplementary figures and images for: 1,25 dihydroxyvitamin D-mediated orchestration of anticancer, transcript-level effects in the immortalized, non-transformed prostate epithelial cell line, RWPE1
Source: BMC Genomics. 2010 Jan 13;11:26. doi: 10.1186/1471-2164-11-26 (PMC2820456; doi:10.1186/1471-2164-11-26)

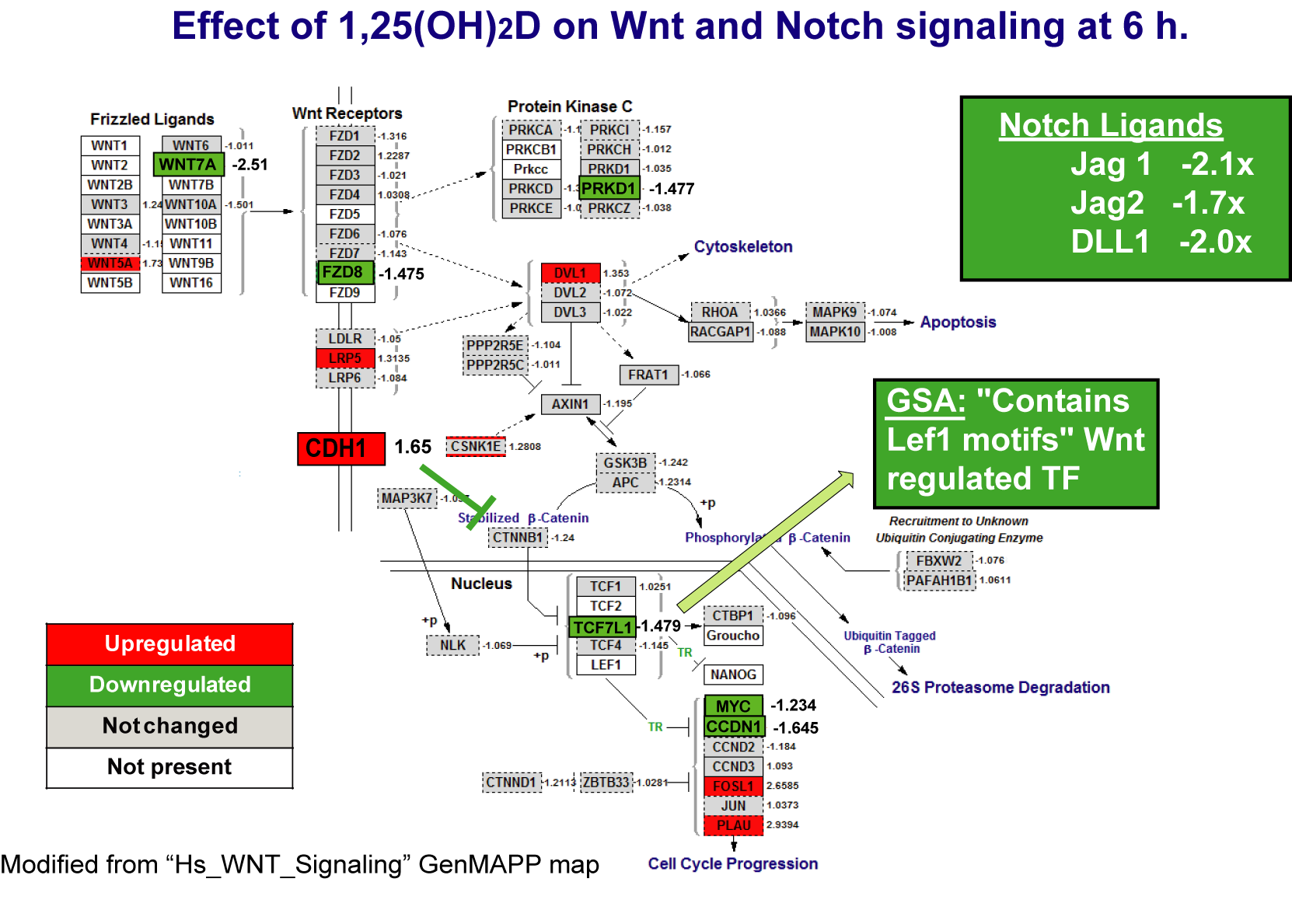

Supplement: Additional file 10 — Effect of 1,25(OH)2D on Wnt and Notch signaling at 6 h. Figure showing the effect of 1,25(OH)2D (100 nM, 6 h) on transcripts controlling Wnt and Notch signaling in RWPE1 cells. Differentially expressed transcripts (SAM, any time point, FDR<5%) were examined by time point for functional changes using GenMAPP and GSA. The GeneMapp local map for Wnt signaling (Hs_WNT_Signaling) was identified as significantly down regulated at 6 h. In addition, a GSA motif geneset (c3 #162) for genes containing Lef1 domains in their promoters (a Wnt pathway targeted transcription factor) was significantly down-regulated. [file 1471-2164-11-26-S10.TIFF]

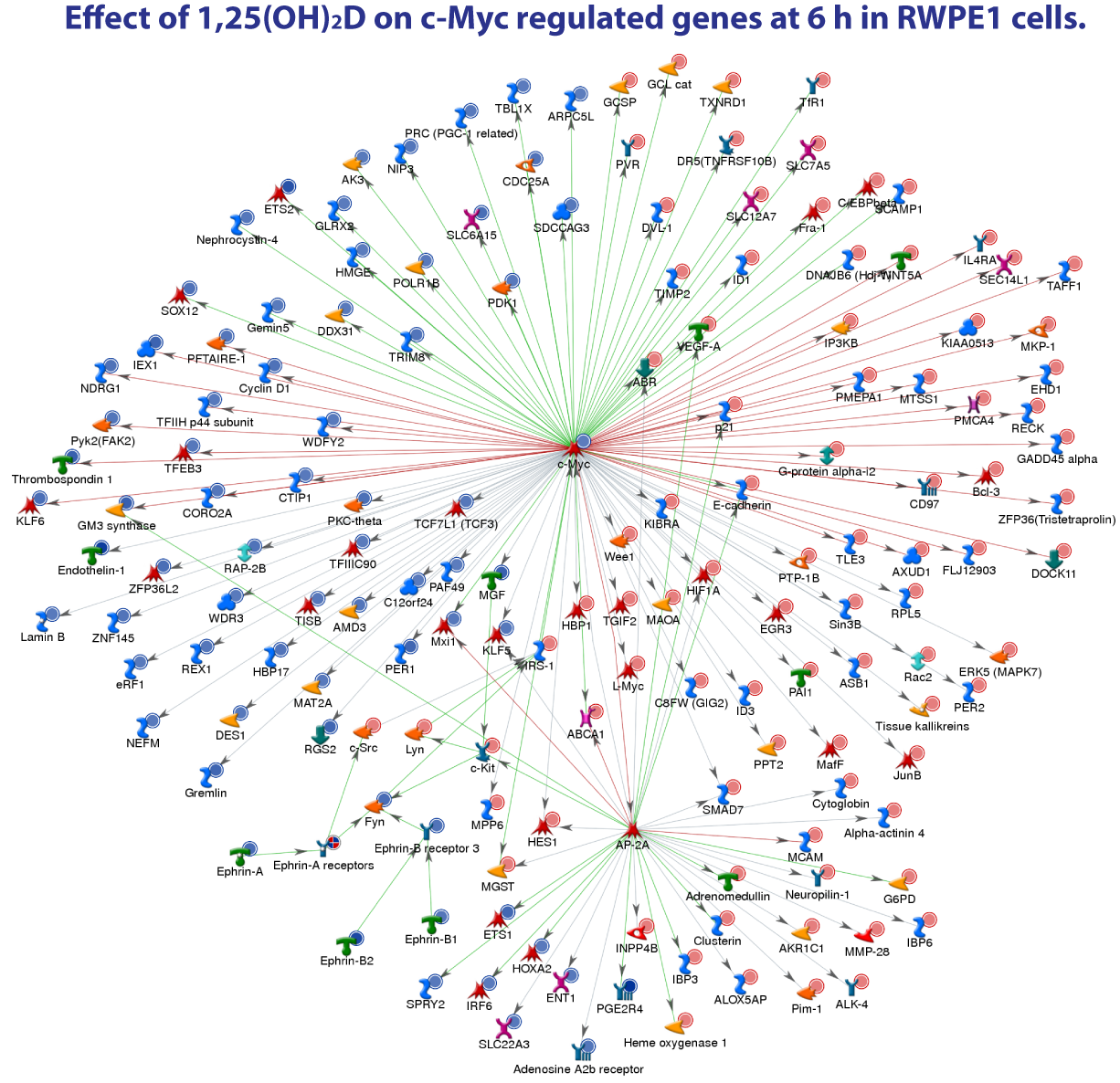

Supplement: Additional file 11 — Effect of 1,25(OH)2D on c-Myc transcriptional activity at 6 h. Image representing effect of vitamin D induced supression of c-Myc on the mRNA level of c-myc target genes. Significantly differentially expressed transcripts at 6 h (SAM, FDR<5%) were analyzed by using Metacore Network analysis (Transcription factor). Up--regulated genes are marked with red circles; down--regulated with blue circles. Arrows are color coded to reflect the known regulatory action between two proteins. Red arrows between proteins indicates a negative regulatory effect, green arrows indicate a positive regulator effect, gray arrows indicate an unspecified regulatory effect. [file 1471-2164-11-26-S11.PNG]

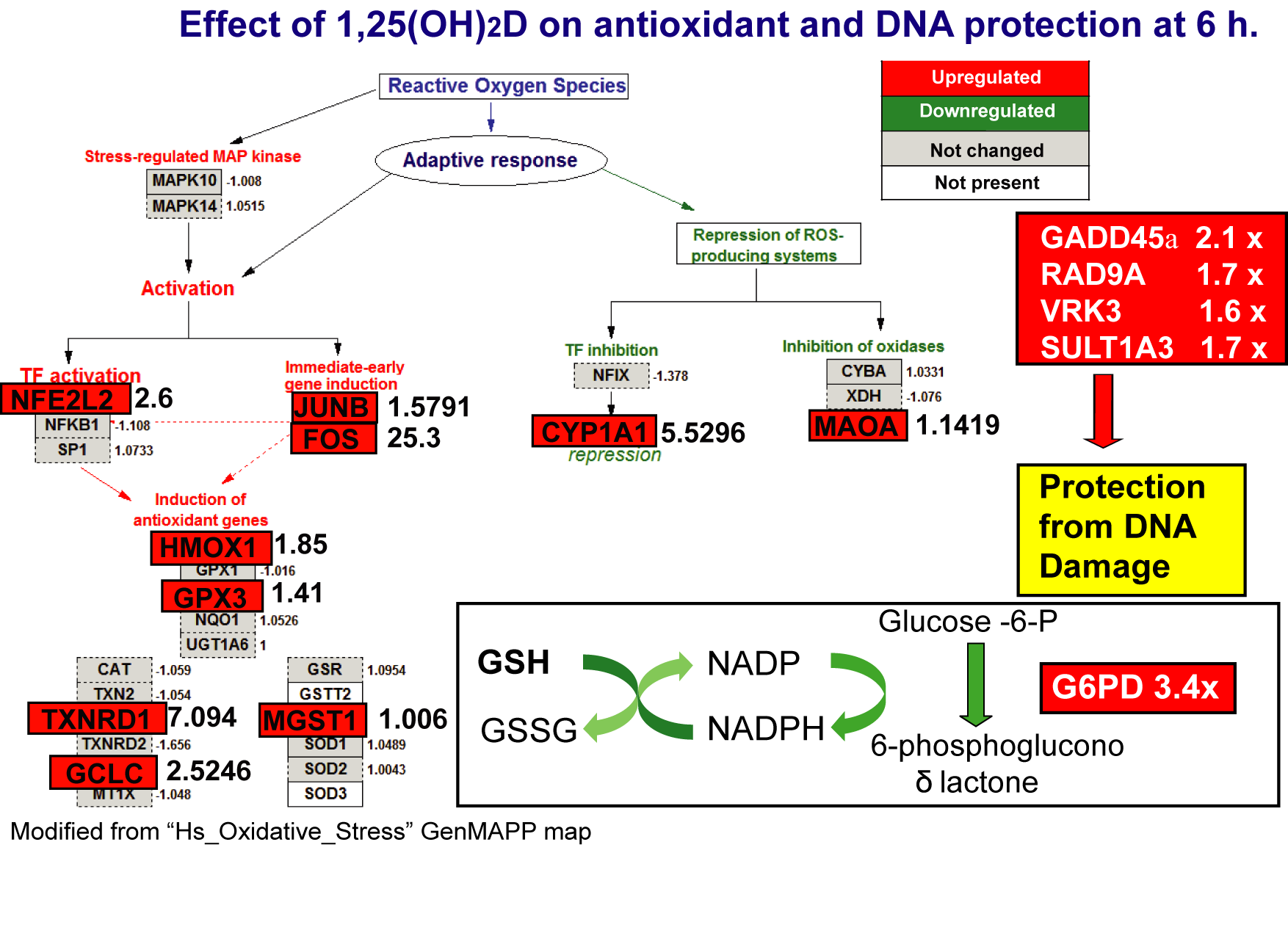

Supplement: Additional file 12 — Effect of 1,25(OH)2D on antioxidant and DNA protection at 6 h. Figure showing the effect of 1,25(OH)2D (100 nM, 6 h) on transcripts controlling antioxidant and DNA repair systems in RWPE1 cells. Differentially expressed transcripts (SAM, any time point, FDR<5%) were examined by time point for functional changes using GenMAPP and GSA. The GenMAPP local map for antioxidant responses to reactive oxygen (Hs_Oxidative_Stress) was identified as significantly up-regulated at 6 h. While not on this map, the up-regulation of G6PD is also relevant as this enzyme system contributes to glutathione production. [file 1471-2164-11-26-S12.TIFF]

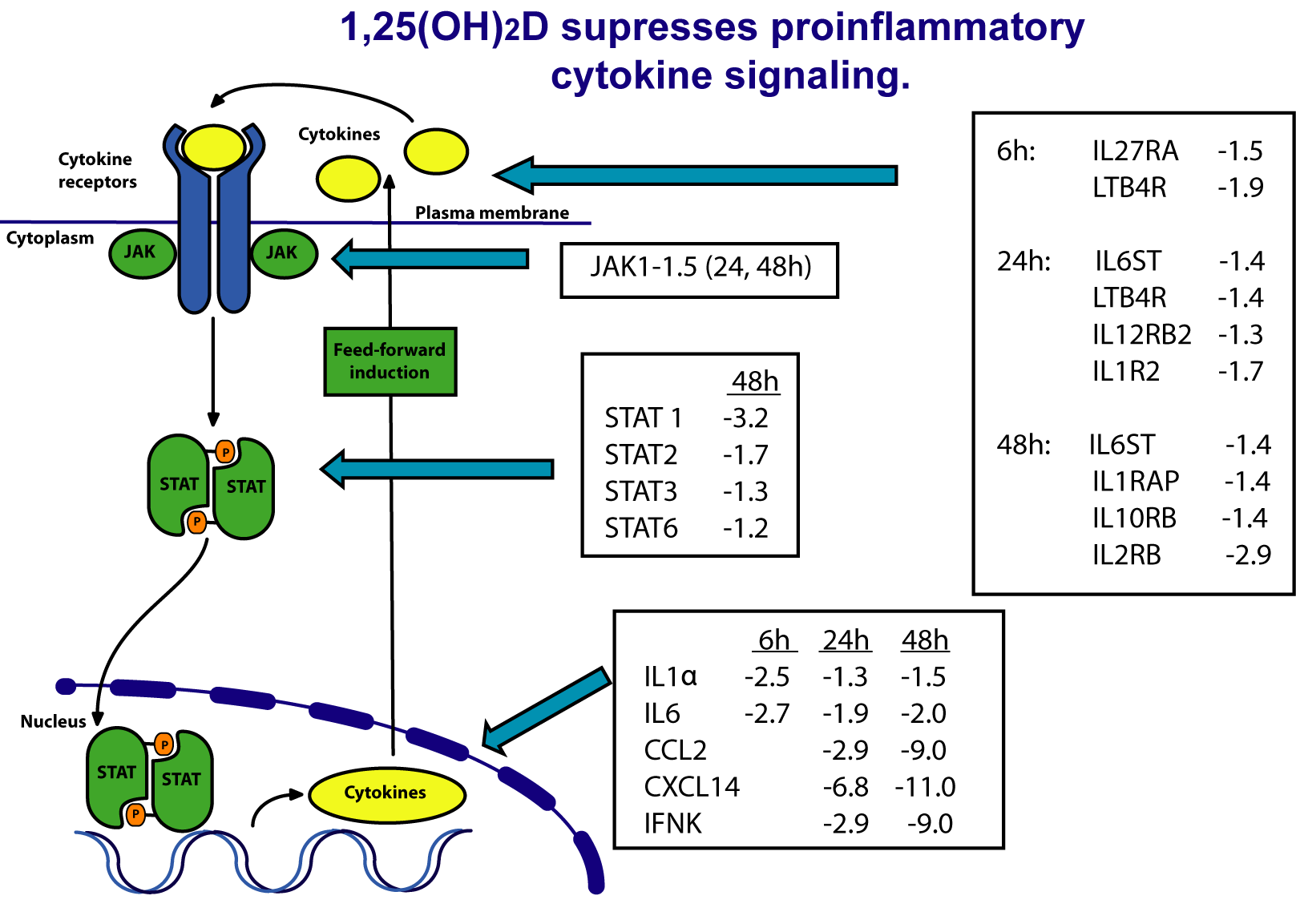

Supplement: Additional file 13 — 1,25(OH)2D suppresses proinflammatory cytokine signaling. Figure representing regulation of transcripts controlling cytokine signaling in RWPE1 cells by 1,25(OH)2D treatment (100 nM). Differentially expressed transcripts (SAM, any time point, FDR<5%) were examined for functional changes using GenMAPP and GSA. A large number of pathways related to the signaling through cytokine pathways were identified as down-regulated. Most of these pathways utilize a JAK-STAT intracellular signaling pathway. A selection of transcripts affected and their relationship to JAK-STAT signaling are shown. [file 1471-2164-11-26-S13.TIFF]

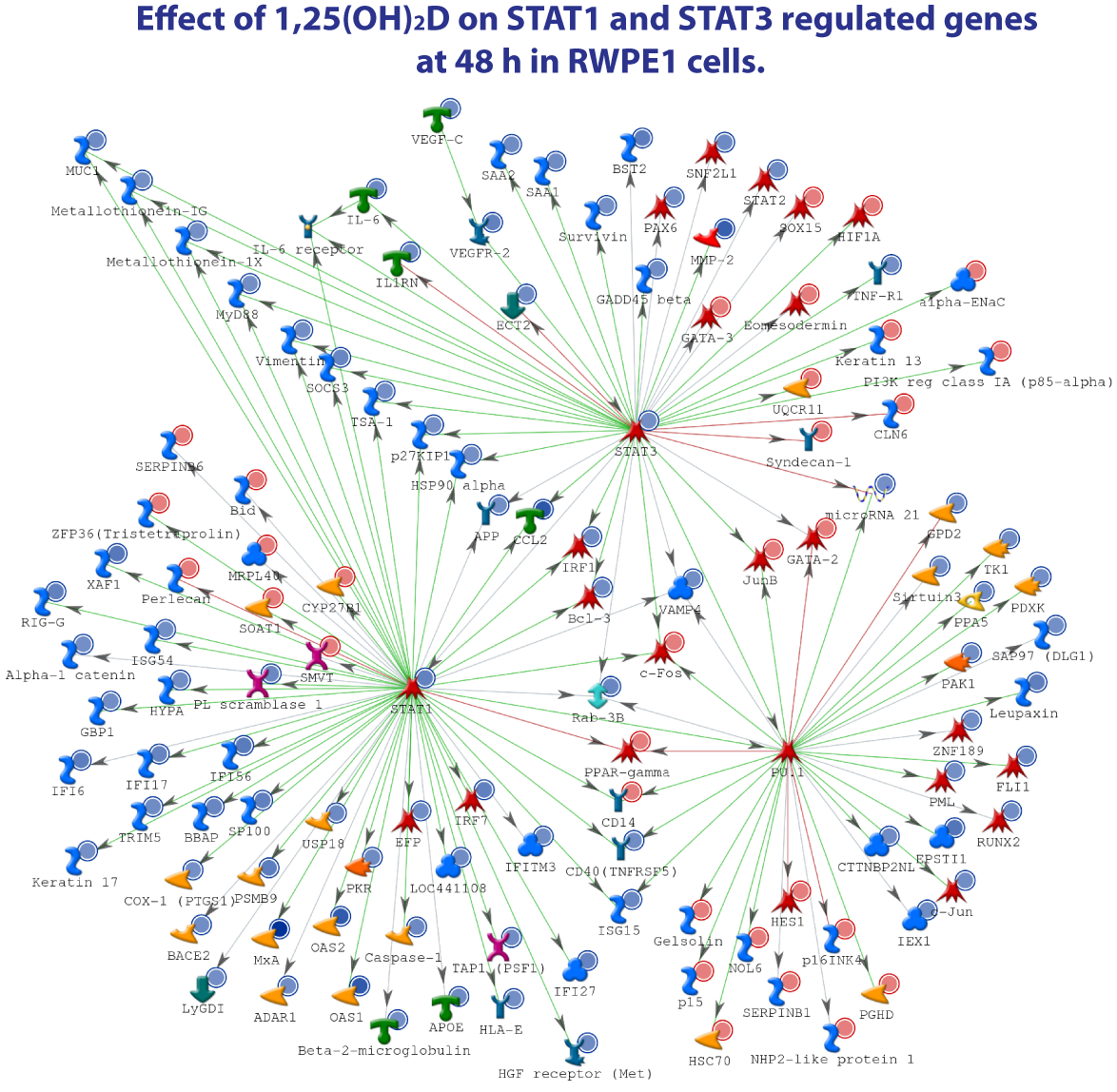

Supplement: Additional file 14 — 1,25(OH)2D suppresses STAT1, STAT3 and PU.1 networks at 48 h. Image representing suppression of transcripts regulated by STAT1, STAT3 and PU.1. Significantly differentially expressed transcripts at 48 h (SAM, FDR<5%) were analyzed by using Metacore Network analysis (Transcription factor). Most of these transcripts are regulated by STAT1 or STAT3. Up--regulated transcripts are marked with red circles; down--regulated transcripts are identified by blue circles. Arrows are color coded to reflect the known regulatory action between two proteins. Red arrows between proteins indicates a negative regulatory effect, green arrows indicate a positive regulator effect, gray arrows indicate an unspecified regulatory effect. [file 1471-2164-11-26-S14.PNG]
