# Supplementary material for: 1,25 dihydroxyvitamin D-mediated orchestration of anticancer, transcript-level effects in the immortalized, non-transformed prostate epithelial cell line, RWPE1
Source: BMC Genomics. 2010 Jan 13;11:26. doi: 10.1186/1471-2164-11-26 (PMC2820456; doi:10.1186/1471-2164-11-26)
Supplement: Additional file 16 — RT-PCR primers. List of RT-PCR primers used for validation of differential expression identified in 1,25(OH)2D-treated RWPE1 cells. [file 1471-2164-11-26-S16.DOC]

RT-PCR primers used for validation of differential expression identified in 1,25(OH)_2_ D-treated RWPE1 cells.

|  | **Gene**  **(Accession ID)** | **Primers for Reverse transcriptional PCR** | **Annealing temperature (T_a_), ⁰C** | **Product size (bp)** |
| --- | --- | --- | --- | --- |
| 1 | CYP24 (NM_000782) | forward ^5’^CTCATGCTAAATACCCAGGTG^3’^  reverse ^5’^TCGCTGGCAAAACGCGATGGG^3’^ | 54.0 | 299 |
| 2 | TRPV6 (NM_018646) | forward ^5’^TTCCTGCGGGTGGAAGACAGGCA^3’^,  reverse ^5’^ACGCAGGTCTCTCCTCAGGGTCCC 3’ | 63.0 | 235 |
| 3 | GAPDH (NM_002046) | forward ^5’^TCACCATCTTCCAGGAGCG^3’^,  reverse ^5’^CTGCTTCACCACCTTCTTGA^3’^ | 54.0 | 571 |
| 4 | IGFBP3 (NM_000598) | forward ^5’^ACCCAGAACTTCTCCTCCGAGTC^3’^,  reverse ^5’^CTGGGAGAGGCTGCCCATACTTA^3’^ | 52.8 | 233 |
| 5 | P2RY2 (NM_002564) | forward ^5’^TGGCGCTCTACATCTTCTTG^3’^,  reverse ^5’^TGCTGCAGTAAAGGTTGGTG^3’^ | 57.1 | 603 |
| 6 | CD14 (NM_000591.2) | forward ^5’^ CGCTCCGAGATGCATGTG^3’^,  reverse ^5’^ AACGACAGATTGAGGGAGTTCAG^3’^ | 59.8 | 52 |
| 7 | TXNRD1 (NM_003330) | forward ^5’^CTTGTGGCCTTTCTGAGGAG^3’^,  reverse ^5’^ CTGCCAAATGTCAGCTTCA^3’^ | 53.7 | 650 |
| 8 | APCDD1 (NM_153000.3) | forward ^5’^ GGAGAAGCAGTACCTTCACCA^3’^,  reverse ^5’^ GACCGATAGATGATCCGACAG^3’^ | 61.0 | 176 |
| 9 | CYP26B1 (NM_019885.2) | forward ^5’^ AGGTCTACCAGCAGTTTGTG^3’^,  reverse ^5’^ AAGTAGTCCTTGCCCTGTG^3’^ | 57.2 | 221 |
| 10 | SEMA3B (NM_004636.2) | forward ^5’^ CAGGAAGGATAGAGGATGG^3’^,  reverse ^5’^ ACCCCTGAGTATAGCTCCTC^3^ | 54.0 | 94 |
| 11 | SEMA3F (NM_004186.2) | forward ^5’^ AGCAGACCCAGGACGTGAG^3’^,  reverse ^5’^ AAGACCATGCGAATATCAGCC^3’^ | 55.0 | 116 |
| 12 | VAV3 (NM_006113.4) | forward ^5’^ GACCTCAGGGAGATGGTGAA^3’^,  reverse ^5’^ GCAGACTTTGCAGGATGTGA^3’^ | 59.8 | 393 |
